# Rare gastrointestinal presentation of systemic mastocytosis, a case report

**DOI:** 10.1016/j.amsu.2022.104196

**Published:** 2022-07-13

**Authors:** Arezoo Eftekhar Javadi, Elham Nazar, Niousha Momeni

**Affiliations:** Department of Pathology, Sina Hospital, Tehran University of Medical Sciences, Iran

**Keywords:** Mastocytosis, Gastrointestinal, Abdominal pain

## Abstract

**Introduction:**

Systemic mastocytosis is a rare disease resulting from infiltration of atypical mast cells in multiple organ systems and present with variety of symptoms. Primary appendiceal and cecal mass with isolated abdominal pain as a presenting feature in systemic mastocytosis have not been reported in literature up to now.

**Case report:**

We described a 69- years-old female with systemic mastocytosis who presented with chronic abdominal pain and recent progression. On imaging of the abdomen and pelvis showed a mass in cecum. The patient underwent surgery and histopathologic evaluation of cecal and appendiceal masses revealed uniform small round cell tumor with eosinophilic cytoplasm admixed with many eosinophils distorting normal colonic mucosal architecture. The neoplastic cells showed positive expression of CD117 and Mast cell tryptase. According to all these considerations systemic mastocytosis was confirmed as the diagnosis.

**Conclusion:**

Isolated abdominal pain and primary large intestinal mass are uncommon features of systemic mastocytosis. This case report informed physicians and pathologists to consider it as one of differential diagnosis.

## Highlights

• Mastocytosis is an uncommon neoplastic infiltration of mast cells in one or multiple organs.
• Isolated abdominal pain and primary large intestinal mass are uncommon features of systemic mastocytosis.
• Mastocytosis reveal common surprising clinical signs that to conclude lead to inadequate treatment. mastocytosis.

## 1 Background

Mastocytosis is an uncommon neoplastic infiltration of mast cells in one or multiple organs. Majority of patients with Mastocytosis have evidence of cutaneous involvement and less of them present with other organs involvement include the spleen, lymph nodes, liver and gastrointestinal (GI) tract [1]. Mastocytosis is a clinically and pathologically heterogenous disease [2]. Mastocytosis is a quite uncommon disorder and underdiagnosed for its nonspecific findings with little data about the epidemiology [3]. Up to now, little is described about the frequency and occurrence of mastocytosis. An overall incidence estimated 0.9 per 100,000 per year [4]. According to new update of World health organization (WHO), Mastocytosis classified in three variants: cutaneous Mastocytosis, systemic Mastocytosis and mastcell sarcoma [5]. Releasing mast cells mediators is the main etiology for presenting clinical manifestations of systemic Mastocytosis, also accumulation and infiltration of mast cells in the tissue introduced as another reason [6]. Diagnosis is based on findings of atypical mast cells infiltration in histopathologic evaluation associated with presence of C-Kit mutation or persistent elevation of serum Tryptase level [5]. If the GI manifestations present as the only symptom, because of its non specificity, diagnosis of systemic Mastocytosis would be extremely difficult and simply ignored. Here we report an unusual case of Mastocytosis presented by isolated abdominal pain and simultaneous cecal and appendiceal masses without any symptoms or signs. This case report has been reported in line with the SCARE Criteria [7].

## 2 Case presentation

**Fig. 1:**
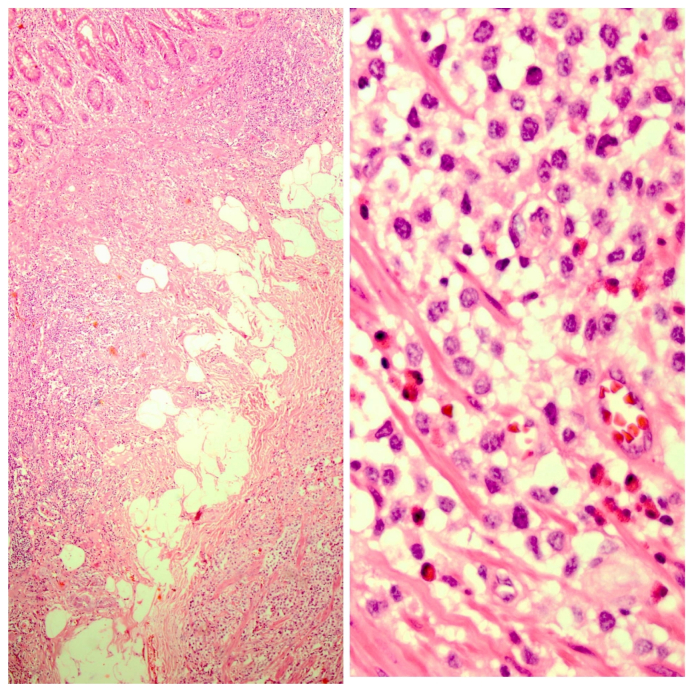
Histopathologic examination showed isolated monotonous infiltration round to oval neoplastic cells admixed with abundant eosinophils in submucosa of intestinal wall (left, H&E X40 and right, H&E X400).

**Fig. 2:**
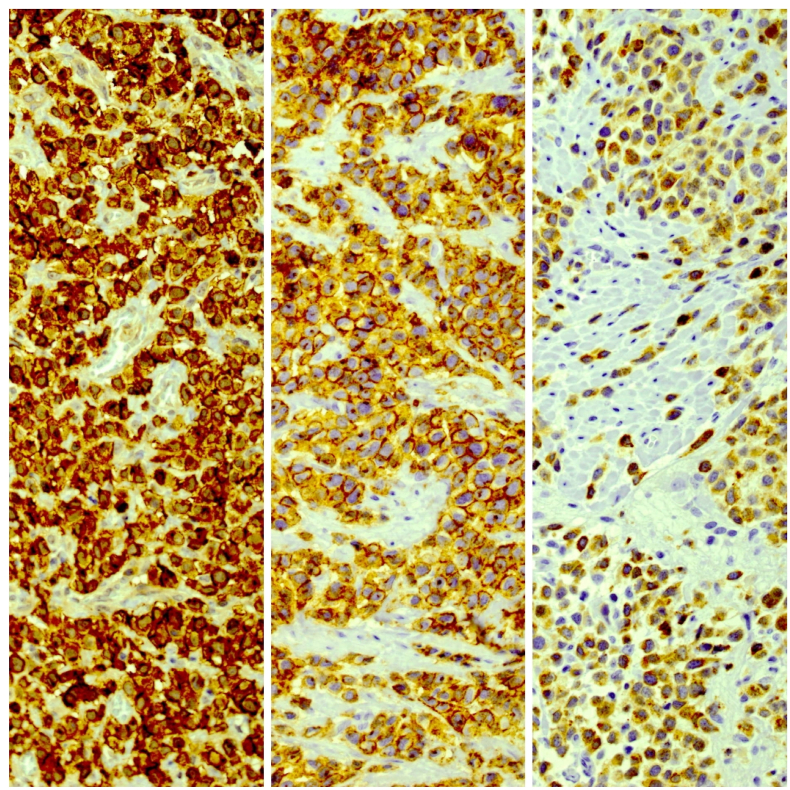
Immunohistochemical staining showed positive CD117 (left), LCA (middle), and tryptase (right) X200.

A 63- years-old woman with chief complaint of chronic abdominal pain and recent worsening referred to surgery department of Sina hospital affiliated to Tehran University of Medical Sciences in November 2019. She didn't have complained any other symptoms such as nausea, vomiting, diarrhea, loss of appetite, weight loss, skin rashes or flushing. In physical examination, vital signs were normal. Abdomen was soft in palpation without any tenderness and appearance abnormalities. Cardiopulmunary, skin and other organ systems examination were unremarkable. She had no familial history, past medical, and past surgical history. Laboratory analysis showed mild leukocytosis, other hematologic, biochemical and urine tests were all unremarkable. On imaging, one mass in cecum was seen. Patient was scheduled for right hemicolectomy and para-aortic lymph node dissection by expert colorectal surgeon. The specimen was received at Sina hospital pathology lab for histopathologic examination consists of a segment of colon, cecum, terminal ileum and appendix. Colon and terminal ileum were 26cm in length and 4.5cm in maximum diameter. On opening a whitish solid submucosal mass was seen in cecum measuring 2x1.5 × 1cm. Also, a 0.7cm whitish lesion was noted in the base of appendix. Microscopic examination for both cecal and appendiceal lesion revealed infiltration of monotonous neoplastic cells with round to ovoid nuclei, eosinophilic cytoplasm, prominent nucleoli and distinct cell boundaries distorting the normal colonic mucosal architecture, also many eosinophils were present admixed with neoplastic cells (Fig. 1). Immunohistochemistry (IHC) staining showed positive result for leukocyte common antigen (LCA), CD117, CD68, Mast cell tryptase and CD99 in tumor cells. Ki67 show proliferative activity in about 30% of neoplastic cells (Fig. 2). The specimen labeled as Para-aortic lymph nodes consists of three pieces of fibrofatty tissue involved by tumor with the same histopathologic features mentioned above. According to microscopic examination and IHC study diagnosis of Mastocytosis was confirmed. After surgery the patient transferred to Intensive Care Unit (ICU) due to critical condition and after few days she expired due to unstable situation.

## 3 Discussion

Mastocytosis is a rare disease resulting infiltration and accumulation of a clonal, neoplastic proliferation of morphologically and immunophenotypically abnormal mast cells in one or multiple organ systems [8] cause different types of clinical manifestations ranging from skin limited symptoms particularly in pediatric cases to aggressive forms with extracutaneous involvement subsequent to organ failure associated with poor prognosis [[9], [10], [11], [12]]. Systemic mastocytosis with multiple organs involvement generally occur in adault patients lacking skin manifestations which are similar to our case. The organ systems which involved in most cases of systemic mastocytosis are bone marrow, liver, spleen, GI tract and lymph nodes [5]. GI symptoms present in up to 80% of patients with systemic mastocytosis and abdominal pain is the most common GI symptom among them. The main etiology for these manifestations is the release of mast cells mediators, although in rare case of advanced systemic mastocytosis, infiltration of mast cells in GI mucosa introduced as the reason [6,[13], [14], [15], [16]]. In our case, the main reason of GI symptoms was masses with mast cells infiltration. According to WHO diagnostic criteria when the major criteria and one minor criteria or at least three minor criteria are satisfied diagnosis of systemic mastocytosis could be made. The major criteria are the presence of multifocal, dense aggregation of at least 15 mast cells in bone marrow biopsy or another extra cutaneous organ. This is the most important part to establish systemic mastocytosis diagnosis while minor criteria determine just the clonal nature or aberrant phenotype of mast cells [5,[17], [18], [19]]. Diagnosis of systemic mastocytois might be hard or underdiagnosed because of its multiple nonspecific manifestations especially when characteristic skin lesion is not present. As we described in our case when GI manifestations are isolated or predominant, diagnosis would be more challenging and simply ignored because of low incidence of systemic mastocytosis and wide range of diagnosis to be mentioned for patient who have simultaneous appendiceal and cecal mass presented with abdominal pain as the only symptom. According to literature review presentation of systemic mastocytosis with primary GI mast cell neoplasm haven't been reported yet. This case report informed physicians and pathologists to consider this diagnosis for patients presented with chronic abdominal pain and large intestinal mass with isolated monotonous cells infiltration on microscopic examination to prevent mismanagement and morbidities.There is no definite treatment for systemic mastocytosis and available treatments are based on symptomatic control by avoidance of mast cell degranulation or inactivation of mast cells mediators. New treatment targets such as Kit mutations and other similar relevant options in neoplastic mast cells have been introduced in recent years [14,[20], [21], [22]]. Although, definite diagnosis for Mastocytosis with unusual presentation is difficult, approaches for diagnosis and treatment devoid of any problem are serious.

## 4 Conclusion

Isolated Mastocytosis in the GI tract is rare but they must be careful in the differential diagnosis of various GI masses. Difficulties in the differential diagnosis might happen in connection with an unexpected setting. They reveal common surprising clinical signs that to conclude lead to inadequate treatment. It is our hope, that with such a report, we are able to reduce misdiagnosis and improve the outcome of these patients.

## Ethical approval

Ethical approval is not needed due to the fact that the treatment of the patient was based on approved options and was not controversial.

## Sources of funding

No source to be stated.

## Authors' contributions

AEJ: major contribution of the idea, study design and revise the paper, EN: pathologist and writing the paper, NM: data collection and follow up.

## Registration of research studies

Name of the registry:
Unique Identifying number or registration ID
Hyperlink to your specific registration (must be publicly accessible and will be checked)

## Guarantor

Elham Nazar is Guarantor of this submission.

## Consent

Written informed consent was obtained from the patient for publication of this case report and accompanying images. A copy of the written consent is available for review by the Editor-in- Chief of this journal on request.

## Provenance and peer review

Not commissioned, externally peer-reviewed.

## Declaration of competing interest

There is no conflict to be declared.
